# Association analysis of insulin-like growth factor-1 axis parameters with survival and functional status in nonagenarians of the Leiden Longevity Study

**DOI:** 10.18632/aging.100841

**Published:** 2015-11-12

**Authors:** Evie van der Spoel, Maarten P. Rozing, Jeanine J. Houwing-Duistermaat, P. Eline Slagboom, Marian Beekman, Anton J.M. de Craen, Rudi G.J. Westendorp, Diana van Heemst

**Affiliations:** ^1^ Department of Gerontology and Geriatrics, Leiden University Medical Center, The Netherlands; ^2^ Netherlands Consortium of Healthy Aging (NCHA), The Netherlands; ^3^ Department of Medical Statistics, Leiden University Medical Center, The Netherlands; ^4^ Section Molecular Epidemiology, Leiden University Medical Center, The Netherlands; ^5^ Department of Public Health, University of Copenhagen, Denmark

**Keywords:** human, familial longevity, IGF-1 axis, survival, functional status

## Abstract

Reduced insulin/insulin-like growth factor 1 (IGF-1) signaling has been associated with longevity in various model organisms. However, the role of insulin/IGF-1 signaling in human survival remains controversial. The aim of this study was to test whether circulating IGF-1 axis parameters associate with old age survival and functional status in nonagenarians from the Leiden Longevity Study. This study examined 858 Dutch nonagenarian (males≥89 years; females≥91 years) siblings from 409 families, without selection on health or demographic characteristics. Nonagenarians were divided over sex-specific strata according to their levels of IGF-1, IGF binding protein 3 and IGF-1/IGFBP3 molar ratio. We found that lower IGF-1/IGFBP3 ratios were associated with improved survival: nonagenarians in the quartile of the lowest ratio had a lower estimated hazard ratio (95% confidence interval) of 0.73 (0.59 – 0.91) compared to the quartile with the highest ratio (p_trend_=0.002). Functional status was assessed by (Instrumental) Activities of Daily Living ((I)ADL) scales. Compared to those in the quartile with the highest IGF-1/IGFBP3 ratio, nonagenarians in the lowest quartile had higher scores for ADL (p_trend_=0.001) and IADL (p_trend_=0.003). These findings suggest that IGF-1 axis parameters are associated with increased old age survival and better functional status in nonagenarians from the Leiden Longevity Study.

## INTRODUCTION

The role of the evolutionarily conserved insulin/insulin-like growth factor (IGF-1) signaling (IIS) pathway in the regulation of lifespan is well documented in worms [1], flies [2], and rodents [3,4]. Genetic mutations that inhibit IIS activation prolong lifespan in these organisms, particularly in the female sex. The involvement of IIS modulation in human longevity is less clear [5]. In agreement with the findings in model organisms, reduced IIS pathway activity was associated with old age survival in sporadic female octogenarians and different cohorts of nonagenarians [6,7] as well as with better cognitive function [8]. Furthermore, centenarians were shown to be enriched for rare IGF-1R mutations associated with IGF-1 resistance [9]. As in mice, mutations causing growth hormone (GH) resistance resulting in low circulating levels of IGF-1 have been reported to confer protection against the development of cancer and diabetes in men [10].

Circulatory IGF-1 is mostly bound to any of six high affinity IGF binding proteins, of which IGF binding protein 3 (IGFBP3) is the most abundant. The IGFBP3 glycoprotein forms a complex with IGF-1 and an acid-labile component and serves as a reservoir of IGF-1 in the circulation. IGF-1 is only biologically active in its free form, which accounts for approximately 1% of total IGF-1. Therefore the IGF-1/IGFBP3 molar ratio is considered a better indicator of IGF-1 bioavailability than total IGF-1. [11,12] Both IGF-1 and IGBP3 are under control of GH [13]. With age, levels of GH decline as do the levels of IGF-1 and IGFBP3 [14]. In contrast to the apparent beneficial effects associated with constitutively low GH/IGF-1 activity discussed above, lower serum IGF-1 levels in humans have also been associated with an increased risk of developing cardiovascular disease and diabetes [15].

In order to identify heritable determinants of longevity we set up the Leiden Longevity Study. This study includes nonagenarian siblings, recruited from 421 Caucasian families based on proband siblings that both exhibit exceptional longevity [16] and their offspring [17]. Using pathway analysis, a significant difference was detected between nonagenarians from the Leiden Longevity Study and a younger age group for the joint effect of genetic variation in the insulin/IGF-1 signaling pathway [18]. In seeming contrast, earlier we reported on the lack of differences in serum levels of IGF-1 axis parameters between middle-aged offspring of familial nonagenarians and controls [19]. However as not all offspring will inherit the favorable genetic predisposition for longevity of their long-lived parent it is unclear to what extent serum levels of IGF-1 axis parameters in middle-aged offspring are reflective of a constitutional phenotype predisposing to longevity. Therefore, in the current study we aim to examine whether circulating levels of IGF-1, IGFBP3 and IGF-1/IGFBP3 molar ratio are associated with old age survival and functional status in nonagenarian siblings from the Leiden Longevity Study.

## RESULTS

### Baseline characteristics

The baseline features of the study population (n=858) are displayed stratified for women (n=528) and men (n=330) in Table 1. The median age of women (93.6 years) was higher than that of men (91.4 years). Women and men also showed significant differences in circulating levels of IGFBP3 and the IGF-1/IGFPB3 molar ratio as well as in scores for cognition, functional status and circulating levels of free triiodothyronine (Table 1).

**Table 1:** Baseline characteristics of the study population

	Women	Men	P-value
Participants (N)	528	330	
Age (years)	93.6 (92.2 – 95.3)	91.4 (90.1 – 93.7)	**<0.001**
Family mortality history score parents	−1.4 (−2.8 – −0.2)	−1.3 (−2.5 – −0.2)	0.94
IGF-1 (nmol/L)	10.1 (7.7 – 13.0)	10.1 (7.6 – 13.2)	0.98
IGFBP3 (mg/L)^†^*	3.2 (0.9)	2.7 (0.8)	**<0.001**
IGF-1/IGFBP3 molar ratio*	0.09 (0.08 – 0.11)	0.11 (0.10 – 0.13)	**<0.001**
Disability (points)			
Mini-Mental State Examination^∼^	25 (21 - 28)	26 (24 – 28)	**0.03**
Activities of Daily Living (ADL)^#^	17 (13 – 19)	19 (17 – 20)	**<0.001**
Instrumental ADL^#^	7 (3 – 11)	10 (6 – 12)	**<0.001**
Non-fasted glucose^•^	6.1 (5.3 - 7.2)	6.1 (5.4 – 7.1)	0.81
Non-fasted insulin	21.0 (11.3 – 36.0)	23.0 (12.0 – 37.0)	0.39
Free triiodothyronine (pmol/L)^†^	4.0 (0.7)	4.1 (0.7)	**0.04**
High sensitivity C-reactive protein (mg/L)^‡^	2.7 (1.3 – 5.4)	3.0 (1.4 – 6.6)	0.31

Unless specified otherwise, data are presented as median with interquartile ranges and analyzed with a non-parametric Median Test.

† data are presented as mean with standard deviation and analyzed with linear regression.

* data available for 527 women and 329 men;

∼ data available for 467 women and 308 men;

# data available for 488 women and 307 men;

• data available for 527 women and 327 men;

‡ data available for 528 women and 329 men

### Association of IGF-1 axis parameters and survival

Nonagenarian siblings were divided over four sex-specific strata according to their circulating levels of IGF-1, IGFBP3 or their IGF-1/IGFBP3 molar ratio. In Table 2, we assessed the relation between quartiles of serum IGF-1 axis parameters and survival, using a left truncated Cox proportional hazards model to correct for the delayed entry into the risk set according to age. Table 2 shows that lower IGF-1/IGFBP3 molar ratios were associated with significantly lower hazard ratios (p for trend = 0.002). There was no interaction between sex-specific quartiles of IGF-1/IGFBP3 molar ratios and sex (p = 0.57). We found a proportional hazard ratio of 0.73 for nonagenarians in the quartile with the lowest IGF-1/IGFBP3 ratio, which is indicative of a 27% higher chance of survival compared to nonagenarians in the quartile with the highest IGF-1/IGFBP3 ratio. Moreover, observed effects did not change during the course of follow-up (data not shown).

**Table 2:** Estimated hazard ratios for sex-specific quartiles of serum IGF-1 axis parameters

	Median (range) women	Median (range) men	Hazard ratio	P-value
IGF-1 (nmol/L)				
Q1	6.3 (3.1 – 7.6)	6.5 (3.6 – 7.5)	0.89 (0.72 – 1.11)	0.30
Q2	8.7 (7.7 – 10.0)	9.0 (7.6 – 10.0)	0.81 (0.65 – 1.00)	**0.05**
Q3	11.4 (10.1 – 12.9)	11.4 (10.1 – 13.0)	0.94 (0.77 – 1.14)	0.52
Q4	15.8 (13.0 – 31.3)	15.3 (13.2 – 30.5)	1 (ref)	
		P for trend	0.15	
IGFBP3 (mg/L)				
Q1	2.2 (0.8 – 2.5)	1.8 (1.0 – 2.0)	1.11 (0.90 – 1.37)	0.35
Q2	2.8 (2.6 – 3.0)	2.3 (2.1 – 2.5)	1.04 (0.85 – 1.26)	0.72
Q3	3.3 (3.1 – 3.6)	2.8 (2.6 – 3.0)	0.99 (0.81 – 1.20)	0.89
Q4	4.2 (3.7 – 6.7)	3.5 (3.1 – 10.1)	1 (ref)	
		P for trend	0.31	
IGF-1/IGFBP3molar ratio				
Q1	0.07 (0.05 – 0.08)	0.09 (0.02 – 0.10)	0.73 (0.59 – 0.91)	**0.005**
Q2	0.09 (0.08 – 0.09)	0.11 (0.10 – 0.11)	0.74 (0.59 – 0.92)	**0.007**
Q3	0.10 (0.09 – 0.11)	0.12 (0.11 – 0.13)	0.87 (0.72 – 1.06)	0.16
Q4	0.13 (0.11 – 0.21)	0.15 (0.13 – 0.29)	1 (ref)	
		P for trend	**0.002**	

Data are presented as estimated hazard ratios with 95% confidence intervals per sex-specific quartiles (Q) of IGF-1, IGFBP3 or IGF-1/IGFBP3 molar ratio as compared to highest quartile, analyzed with cox regression adjusted for family relationship.

### Association of IGF-1/IGFBP3 molar ratio and functional status

Next we assessed the relation between quartiles of serum IGF-1 axis parameters and available baseline measures of functional status and health. Table 3 shows that lower IGF-1/IGFBP3 molar ratios in nonagenarians were associated with less physical disability. Compared to nonagenarians in the quartile with the highest IGF-1/IGFBP3 ratios, those in the quartile of the lowest IGF-1/IGFBP3 molar ratios had higher mean (95% CI) scores for both Activities of Daily Living (ADL) (16.8 (16.3 – 17.4) vs 15.2 (14.5 – 15.9), p for trend = 0.001) and Instrumental Activities of Daily Living (IADL) (8.0 (7.4 – 8.7) vs 6.9 (6.3 – 7.5), p for trend = 0.003). The Mini-Mental State Examination (MMSE) did not significantly differ across quartiles. Interestingly, lower IGF-1/IGFBP3 molar ratios were associated with lower levels of non-fasted insulin.

**Table 3:** Baseline characteristics for quartiles of IGF-1/IGFBP3 molar ratio

	Q1	Q2	Q3	Q4	P for trend
**Demographics**					
Participants (N)	213	213	215	215	
Men (N, %)	82 (38.5)	82 (38.5)	83 (38.6)	82 (38.1)	0.95
Age (years)	93.5 (93.1 – 93.8)	93.3 (93.0 – 93.7)	93.4 (93.0 – 93.7)	93.1 (92.8 – 93.5)	0.27
**Disability (points)**					
Mini-Mental State Examination^∼^	24.4 (23.7 – 25.2)	24.5 (23.7 – 25.2)	24.5 (23.8 – 25.1)	23.6 (22.8 – 24.4)	0.11
Activities of Daily Living (ADL)^#^	16.8 (16.3 – 17.4)	16.4 (15.7 – 17.1)	16.2 (15.6 – 16.8)	15.2 (14.5 – 15.9)	**0.001**					
Instrumental ADL^#^	8.0 (7.4 – 8.7)	8.2 (7.6 – 8.8)	7.6 (7.0 – 8.1)	6.9 (6.3 – 7.5)	**0.003**					
**Serum parameters**					
Non-fasted glucose^•^	6.2 (5.9 – 6.4)	6.4 (6.2 – 6.6)	6.4 (6.2 – 6.6)	6.4 (6.2 – 6.7)	0.10
Non-fasted insulin	16.3 (14.4 – 18.5)	20.7 (18.5 – 23.2)	20.9 (18.7 – 23.5)	23.3 (20.9 – 25.9)	**<0.001**					
Free triiodothyronine (pmol/L)	4.0 (3.9 – 4.1)	4.1 (4.0 – 4.2)	4.1 (4.0 – 4.2)	4.1 (4.0 – 4.2)	0.23
High sensitivity C-reactive protein (mg/L)^‡^	2.7 (2.3 – 3.2)	3.2 (2.7 – 3.8)	2.6 (2.2 – 3.1)	3.1 (2.6 – 3.7)	0.64
**Family mortality history score**					
Family mortality history score parents	−1.7 (−1.9 – −1.4)	−1.8 (−2.1 – −1.6)	−1.5 (−1.8 – −1.3)	−1.5 (−1.8 – −1.3)	0.22

Unless specified otherwise, data are presented as mean with 95% confidence interval, analyzed with linear regression adjusted for age and family relationship except for the variables sex, age and family mortality history score parents).

∼ data available for 774 participants (197 in Q1, 193 in Q2, 189 in Q3, 195 in Q4);

# data available for 793 participants (192 in Q1, 196 in Q2, 200 in Q3, 205 in Q4);

• data available for 852 participants (212 in Q1, 212 in Q2, 213 in Q4);

‡ data available for 855 participants (212 in Q1)

### Family mortality history score

Previously, we had calculated a family mortality history score describing the mortality of the parents of the nonagenarian siblings [20]. To assess whether a lower IGF-1/IGBP3 molar ratio is a feature of familial longevity, we compared the family mortality history score across quartiles of IGF-1/IGFBP3 molar ratio. Table 3 shows that lower IGF-1/IGFBP3 molar ratios in nonagenarians siblings were not associated with a lower family mortality history score (i.e. lower than expected mortality of the parents of the nonagenarian siblings).

## DISCUSSION

This study aimed to explore the association of circulating IGF-1 axis serum parameters with survival and functional status in nonagenarian siblings from the Leiden Longevity Study. First we demonstrated that lower IGF-1/IGFBP3 molar ratios conferred a survival benefit at the age of ninety years or older. Secondly, lower IGF-1/IGFBP3 molar ratios were associated with better functional status at the age of ninety years or older.

Our outcomes support the recent observation that low IGF-1 levels predict survival in exceptionally long-lived humans [21]. Not only do we confirm that lower IGF-1 axis serum parameters are related with survival in the oldest old, but we also demonstrate that lower IGF-1 axis serum parameters are associated with better functional status. A large number of studies have reported an association between reduced IIS activity and longevity in various model organisms as well as in human studies showing life extending effects of reduced IGF-1 signaling [1-4,7]. In these studies lifespan extending effects were mostly confined to females, unlike the results presented here. In contrast with our findings, Paolisso *et al*. observed higher serum IGF-1/IGFBP3 ratios in healthy centenarians when compared to aged controls [22]. Another study showed a higher prevalence of heterozygous mutations in the IGF-1R in Ashkenazi Jewish centenarians compared to controls with concomitant higher serum IGF-1 levels [9]. Moreover they found a sex-specific increase in serum IGF-1 associated with a smaller stature in female offspring of centenarians, suggesting the involvement of reduced IGF-1R activity in human longevity [9]. Unfortunately, anthropometric data were not available in our current study. Therefore we cannot draw firm conclusions as to how the IGF-1/IGFBP3 ratios correlate with the underlying IGF-1 signaling activity in our study population.

It has been suggested that low levels of IGF-1 and/or reduced IGF-1 bioavailability form part of a survival response that can be constitutively active in long-lived individuals as well as elicited by diverse forms of stress, including metabolic stress, genotoxic stress and inflammation [23]. In hospitalized elderly patients, frailty, impairments and mortality were associated with a distinct biomarker signature that comprised higher levels of inflammatory markers and lower levels of growth factors and anabolic hormones, including IGF-1 and free triiodothyronine [24]. In our study, levels of CRP and free triiodothyronine did not differ across quartiles of the IGF-1/IGFBP3 molar ratios, while functional status was better in the quartile with the lowest IGF-1/IGFBP3 ratio, in line with the observed survival benefit.

A limitation of our study is that information about medical history, medication use and specific causes of death is lacking. Previous research suggested that enhancement of insulin sensitivity was a key mediator of the increased longevity of hypopituitary, GH- resistant, and calorie-restricted animals [25]. Moreover, preserved insulin sensitivity was also shown to be a key phenotype of human longevity, both at middle age [26] and at extremely high ages [27]. Previously, we observed lower fasted levels of glucose and insulin when the offspring of the included nonagenarians were compared to an age-matched control group [28]. It is a limitation of the current study that all blood parameters, including IGF-1, IGFBP3, glucose and insulin were determined in non-fasted samples collected at a random moment of the day and that data on insulin sensitivity and food intake were lacking. Circulatory levels of IGF-1 will decrease while levels of its binding proteins (notably IGFBP1) will increase in response to reductions in food intake, in particular the intake of protein [29]. Nevertheless, our finding that lower IGF-1/IGFBP3 molar ratios were associated with relatively lower circulating levels of insulin is suggestive of better insulin sensitivity and in accordance with other recent human data [30]. IGF-1 has structural and functional homology with insulin, and it has been suggested that insulin resistance might lead to increased IGF-1 bioavailability to compensate for reduced insulin action. Another limitation of our study is its cross sectional design which precludes causal inference of the observed associations.

Because we found that lower IGF-1/IGFBP3 ratios were associated with better old age survival in nonagenarians of the LLS, we assessed whether nonagenarian IGF-1/IGFBP3 ratios were associated with a family mortality history score describing the mortality of the parents of the nonagenarian siblings. Our findings on lack of association between nonagenarian IGF-1/IGFBP3 ratios and family mortality history score are in accordance with the lack of difference in IGF-1 axis parameters, including IGF-1, IGFBP3 and the IGF-1/IGFBP3 molar ratios previously observed between middle-aged offspring of familial nonagenarians and controls. These disparate results suggest that the possible benefits of low IGF-1/IGFBP3 ratios may differ according to age and birth cohorts. One of the possible explanations is that with advancing age, the IGF-1/IGFBP3 ratio changes and that those individuals that adapt to a lower IGF-1/IGFBP3 ratio have a survival advantage in old age. Another possibility is selective survival of participants with constitutively lower IGF-1/IGFBP3 ratios. It is possible that selective advantage of variation in the IIS pathway may only become detectable at advanced ages. In line, the association between *FOXO3A* and longevity was for example found to be stronger in centenarians than in nonagenarians [31]. In our study, a considerable percentage of included nonagenarians (23.8%) also reached an age of 100 years or more. Amongst others, another possible explanation for these contrasting observations could be differences in imprinting of IGF-1 axis genes, reflecting historical differences in maternal nutrition between generations [32].

In conclusion, we showed that in nonagenarian siblings IGF-1/IGFBP3 molar ratios are associated with better survival and functional status. These preliminary findings support the involvement of IGF-1 signaling in modulating human longevity.

## MATERIALS AND METHODS

In the Leiden Longevity Study, 421 families were recruited consisting of long-lived Caucasian siblings together with their offspring and the partners thereof. For the current study, data on IGF-1 and IGFBP3 levels were available for 858 of the 944 nonagenarian participants from the Leiden Longevity Study. After a median follow-up time of 3.4 years (range 0 – 11.5 years), 797 individuals (92.9%) had died. The Medical Ethical Committee of the Leiden University Medical Center approved the study and informed consent was obtained from all subjects. For details on enrollment please see previous publications [17,19].

All serum measurements were performed with fully automated equipment. For IGF-1, IGFBP3, and insulin the Modular E170 was used, for glucose, high sensitivity C-reactive protein (hsCRP) and free triiodothyronine the Cobas Integra 800 was used, both from Roche, Almere, the Netherlands. The coefficients of variation of these measurements were all below 5%.

Global cognitive function was assessed with the Mini-Mental State Examination (MMSE) and functional status was assessed by Activities of Daily Living (ADL) and Instrumental Activities of Daily Living (IADL) scales as described previously [33]. ADL disability scores range from 0 points (fully dependent in all activities) to 20 points (fully independent in all activities). IADL disability scores range from 0 points (fully dependent in all activities) to 14 points (fully independent in all activities). MMSE scores range from zero points (very severe cognitive impairment) to 30 points (optimal cognitive function).

For each parent we computed the sex and birth cohort cumulative hazards using the life tables of the Dutch population. Note that since both parents are deceased one minus the cumulative hazard equals the martingale residual. The martingale residual is defined as the difference between the event status (0 if alive, 1 if deceased) and the cumulative hazard at the observed age (current age or age at death). The sum of the martingale residuals measures the deviation of survival of the parents with respect to their birth cohort. Therefore negative values indicate excess survival and positive values indicate excess mortality.

Nonagenarians were divided over four sex-specific strata according to their circulating levels of IGF-1, IGFBP3 or their IGF-1/IGFBP3 molar ratio. The association between quartiles of IGF-1 axis parameters and baseline characteristics of nonagenarians was assessed using a linear mixed model corrected for correlation of sibling data using robust standard errors clustered on family number. Distributions of continuous variables were examined for normality and logarithmically transformed when appropriate. Survival analyses were performed with a left truncated Cox proportional hazards model to correct for the delayed entry into the risk set according to age and the model was corrected for correlation of sibling data using robust standard errors clustered on family number. The Statistical Package STATA (“statistics and data”) for Windows, version 12.0 SE, and the Statistical Package for the Social Sciences (SPSS) for Windows, version 20.0, were used for data analysis.
